# Development and clinical application of CAPAH: a long-read sequencing approach for accurate second-tier screening of phenylketonuria in newborns

**DOI:** 10.1186/s13023-026-04537-6

**Published:** 2026-08-12

**Authors:** Shuyuan Xue, Ziyi Feng, Jingying Zhu, Xi Chen, Weibo Huang, Pei Liu, Qi Chen, Guifeng Ding

**Affiliations:** 1 Urumqi Maternal and Child Health Hospital, Urumqi, China; 2 Xinjiang Clinical Research Center for Perinatal Diseases, Urumqi, China; 3 School of Public Health, Shihezi University, Shihezi, China; 4 School of Public Health, Xinjiang Medical University, Urumqi, China

**Keywords:** Phenylketonuria, Long-read sequencing, *PAH*, Newborn screening, Second-tier screening

## Abstract

**Background:**

Phenylketonuria (PKU) is a common inherited metabolic disorder in newborns. Early diagnosis and timely intervention are essential to prevent neurodevelopmental impairment. Traditional newborn screening primarily relies on measuring blood phenylalanine (Phe) levels, which can lead to both false-positive and false-negative results. Long-read sequencing (LRS) offers high coverage and long-fragment resolution, enabling comprehensive detection of *PAH* gene variants and potentially improving PKU screening and diagnosis.

**Method:**

We developed a comprehensive *PAH* gene sequencing technology (CAPAH) based on the HiFi LRS platform, and applied it to the second-tier screening for PKU in newborns. First, CAPAH was validated for consistency with traditional NGS, MLPA, and Sanger sequencing in 20 samples with known genotypes. Subsequently, a retrospective analysis of 200 newborns with positive or borderline primary screening results was performed to assess the impact of CAPAH on variant detection, genotype-phenotype associations, and second-tier screening performance.

**Results:**

CAPAH achieved complete consistency (100%) with traditional methods in the 20 validation samples. It enabled direct determination of cis-trans configurations and precise localization of large deletion breakpoints. Among 200 newborns, CAPAH identified 103 newborns with biallelic variants, 47 carriers, and 50 negative individuals, reducing the recall rate from primary screening by 48.5% (97/200). Among 131 newborns with negative or excluded PKU based on rescreening blood Phe levels, CAPAH detected 40 newborns carrying compound heterozygous variants, preventing approximately 20% of potential missed detections. In the 103 newborns with biallelic variants, CAPAH identified 64 *PAH* variants, including 47 missense, eight splice-site, four nonsense, and five deletion variants. Genotype-phenotype analysis revealed that the proportion of severe biallelic PKU-related variants (classic and mild PKU) was significantly higher in the rescreen-positive group than in the rescreen-negative group (25.00% vs. 14.29%). Homozygosity for c.158G > A or its combination with PKU- or mild hyperphenylalaninemia (MHP)-related variants was associated with blood Phe levels within the MHP range (2–6 mg/dL). When c.158G > A was combined with PKU-related variants, rescreening Phe levels were significantly elevated (*p* < 0.01).

**Conclusion:**

CAPAH enables comprehensive detection of the *PAH* gene, including complex variants and cis–trans configurations, thereby improving the accuracy of second-tier PKU screening in newborns and reducing unnecessary recalls. This approach provides an efficient and practical molecular diagnostic tool for newborn screening of inherited metabolic disease.

**Supplementary Information:**

The online version contains supplementary material available at https://doi.org/10.1186/s13023-026-04537-6.

## Background

Phenylketonuria (PKU) is an autosomal recessive inherited amino acid metabolic disorder caused by pathogenic variants in the phenylalanine hydroxylase (PAH) gene. The incidence in China is approximately 1/15,924, with a higher incidence in northern China compared with southern regions [1]. PKU patients are typically asymptomatic during the neonatal period. However, three to four months after birth, due to the reduced or absent activity of PAH, phenylalanine (Phe) accumulates excessively in the blood. This accumulation leads to progressive clinical manifestations such as intellectual development delay, epilepsy, and a characteristic “mousy” odor in sweat and urine [2]. Without timely intervention, affected newborns may develop severe neurodevelopmental impairment or even permanent intellectual disability [3].

Newborn screening (NBS) for PKU has been widely implemented worldwide, aiming to detect affected newborns early and initiate timely treatment to prevent brain damage and other complications, thereby ensuring normal growth and development. Since Guthrie introduced the bacterial inhibition assay suitable for large-scale screening of hyperphenylalaninemia (HPA), the implementation of PKU screening has steadily expanded and become an essential public health measure [4, 5]. In China, the first round of PKU screening primarily relies on fluorometric assays or tandem mass spectrometry (MS/MS) to measure Phe concentrations in dried blood spots (DBS). A Phe concentration > 120 µmol/L, or a Phe/Tyr ratio > 2.0, prompts recall for a second blood draw to confirm the result. If the retest result remains positive, further differential diagnostic tests, including pterin analysis, erythrocyte dihydropteridine reductase activity measurement, and the tetrahydrobiopterin (BH4) loading test, are required [6]. This multi-step screening process (comprising initial screening, rescreening, and confirmation/exclusion) is time-consuming and costly. Moreover, biochemical screening results can be interfered with by factors such as prematurity or blood transfusion, leading to a high risk of false-positives and false-negatives results [7]. These inaccuracies may cause unnecessary recalls, misdiagnoses, or delays in treatment, wasting medical resources.

Although *PAH* variant analysis is not mandatory for the initial management of newborns with elevated Phe levels detected by NBS, molecular confirmation is essential for definitive diagnosis, particularly in cases that remain inconclusive after the above differential tests [8]. For initially positive samples, performing genetic testing directly on the residual DBS (without recalling the newborn) could reduce the false-positive rate and prevent misdiagnosis or missed diagnoses caused by biochemical interference during testing [9]. Traditional genetic testing methods mainly use multiplex ligation-dependent probe amplification (MLPA) to detect deletion/duplication variants and Sanger sequencing to detect point variants [10, 11]. However, this workflow is labor-intensive and not ideal for large-scale clinical use. Furthermore, the probe regions in the MRC-Holland MLPA kit P055-CL overlap with *PAH* variant hotspots in the Chinese population, increasing the risk of false-positive interpretations [12]. Next-generation sequencing (NGS), which is widely used in clinical practice, can identify PKU-related variants by sequencing multiple genes or the entire genome. However, large deletions still require supplementary MLPA analysis, which increases turnaround time and costs [13]. In addition, when two or more variants are detected, neither MLPA, Sanger sequencing, nor NGS can determine whether they are in cis or trans configurations, which must be inferred from pedigree analysis. This dependency prolongs the diagnostic process and limits testing in cases lacking parental samples. Despite the combined application of Sanger sequencing, NGS, MLPA, and qRT-PCR, the overall diagnostic yield remains suboptimal, and ~ 5% of patients still lack a confirmed molecular diagnosis [14].

In contrast, long-read sequencing (LRS) based on the PacBio platform offers distinct technical advantages, including ultra-long reads, no base bias, and the ability to accurately sequence complex regions such as GC-rich or repetitive sequences. LRS has been increasingly applied in genetic disease testing, but its utility in newborn screening for PKU has not been systematically evaluated [15]. The *PAH* analyzing method based on LRS can not only identify all reported variants, but also detect large-fragment variants due to read lengths exceeding 10 kb. Its lack of GC bias ensures uniform coverage across the GC-rich exon 7 of *PAH*, improving the precision of variant detection [16]. Moreover, when multiple variants are present within a single long-read amplicon, LRS enables direct phasing of variants (cis/trans determination) without requiring parental samples. This feature significantly shortens diagnostic turnaround time, reduces costs, and prevents unnecessary recalls, allowing timely clinical intervention and optimized resource utilization.

Based on the PacBio sequencing platform, we developed an LRS method (CAPAH) that covers all exonic and adjacent intronic regions of *PAH*. This method was applied to newborns with positive initial PKU screening results to evaluate its feasibility and clinical advantages as a " second-tier screening” method.

## Methods

### Participants

A total of 220 newborns who underwent PKU screening at the Department of Prenatal Diagnosis Center, Urumqi Maternal and Child Health Hospital, from January 2019 to December 2024 were included in this study. Among them: (1) Twenty retrospective samples from newborns previously diagnosed with PKU through genetic analysis were used for the methodological validation of the CAPAH assay; (2) Seventy-five retrospective samples from newborns with borderline results in the initial PKU biochemical screening (blood Phe concentration of 1.6-2 mg/dL) and 125 retrospective samples from newborns with positive results in the initial PKU biochemical screening (blood Phe concentration > 2 mg/dL) were included in an exploratory study to evaluate the feasibility of CAPAH as a second-tier screening method for PKU in newborns. This study was approved by the Medical Ethics Committee of Urumqi Maternal and Child Health Hospital (Ethical Approval Number: XJFYLL2019035). Written informed consent was obtained from the parents of all newborn participants.

### Traditional screening and diagnostic procedures for PKU

(1) Sample collection: Heel-prick blood samples were collected from newborns 72 h after birth following full feeding. DBS were prepared for neonatal screening. (2) Screening methods and procedures: Blood Phe concentration was measured using the ninhydrin fluorometric method with the Neonatal Phenylalanine Kit (Ani Labsystems Ltd, Finland). Fluorescence was detected with a time-resolved fluorometer (Wallac 1420 VICTOR2D multicounter 1420, PerkinElmer). An initial screening result was considered positive when the Phe concentration exceeded 2.0 mg/dL. Newborns with positive results were recalled for resampling and retesting. If the rescreening Phe concentration remained > 2.0 mg/dL, the newborn was considered a suspected PKU patient and subjected to confirmatory testing. Confirmatory biochemical analyses included quantitative determination of blood Phe/Tyr ratios, urinary pterin profiling, and erythrocyte dihydropteridine reductase (DHPR) activity measurement. A clinical diagnosis of PKU was then confirmed or excluded accordingly. Because the ninhydrin fluorometric method measures relative rather than absolute Phe concentrations, poor DBS quality or technical errors may result in falsely low readings, leading to missed diagnoses. Therefore, in our center, newborns with borderline initial screening results (blood Phe concentration of 1.6-2 mg/dL) were also included in the recall and rescreening process. (3) Genetic diagnosis: Genomic DNA was extracted using a QIAGEN DNA extraction kit (QIAGEN, Germany). Pathogenic variants associated with PKU were detected using next-generation sequencing (NGS). Sanger sequencing was performed to verify the identified variants and to confirm their parental origin for segregation analysis.

### Development of the CAPAH assay

**Fig. 1:**
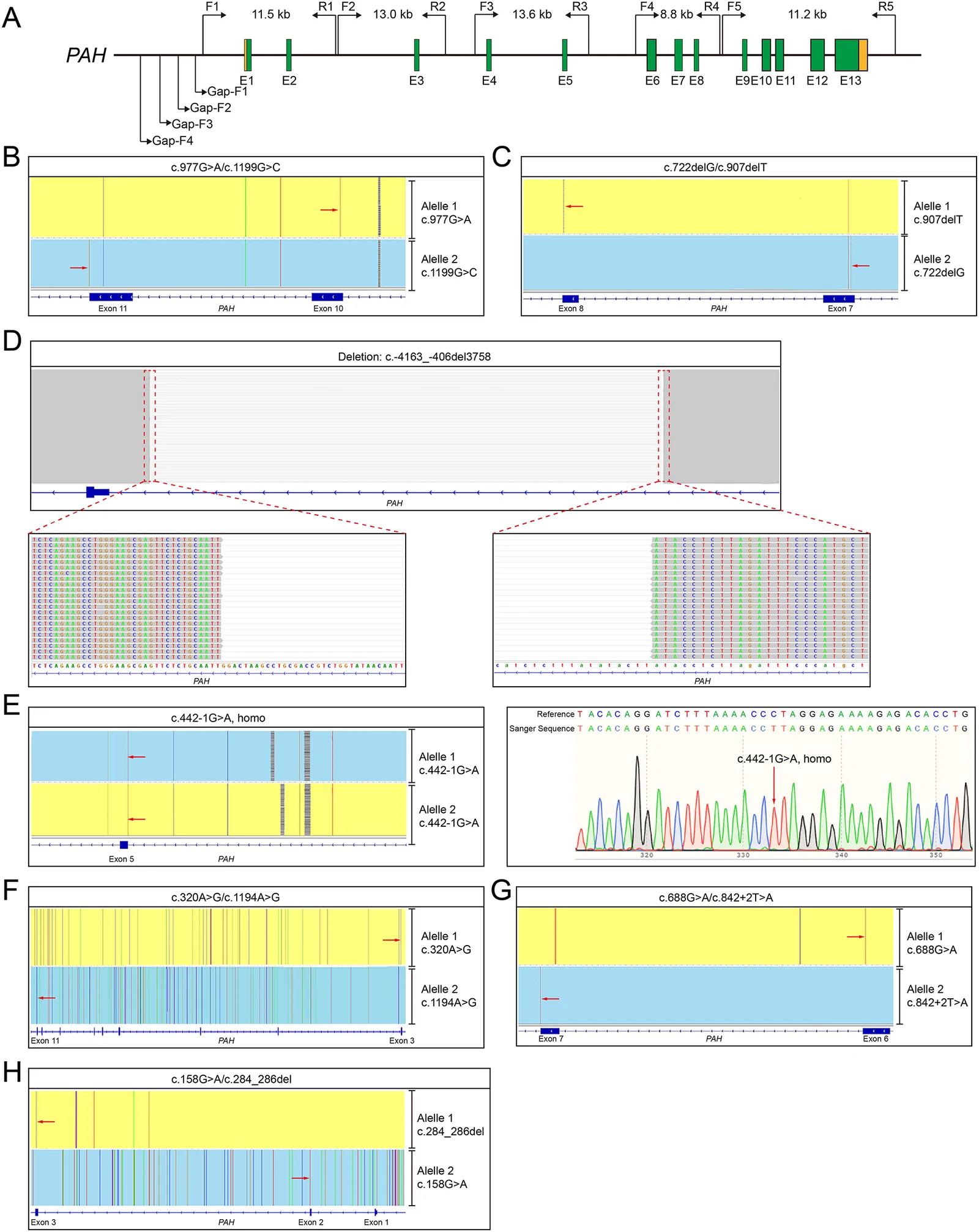
Establishment, validation, and application of the CAPAH assay. **A**. Design of long-range PCR primer pairs covering the *PAH* gene. **B**-**H**. Integrative Genomics Viewer (IGV) plots (and Sanger sequencing for panel E) showing representative *PAH* variants detected by CAPAH. Variants are indicated by red arrows

The *PAH* gene spans approximately 90 kb (hg38 chr2:102,836,885 − 102,917,603) [17] and comprises 13 exons [18]. To achieve complete coverage of the *PAH* coding region, five long-range PCR (LR-PCR) amplicons were designed (Fig. 1A). Primers were optimized to generate amplification products of comparable sizes (8.8 to 13.6 kb) to minimize variability in template loading and sequencing efficiency. Additionally, four GAP-PCR primer sets (GAP-F1-F4) were designed to target the 5’-UTR of PAH in order to detect potential deletion variants in this region. Multiplex LR-PCR was performed on DBS DNA using the KOD FX Neo DNA polymerase (TOYOBO). The integrity of the genomic DNA from these retrospective DBS samples was demonstrated via an agarose gel electrophoresis (Fig. S1), which clearly shows both the high-molecular-weight genomic DNA and the corresponding LR-PCR amplicons. The optimized PCR cycling conditions for fragment amplification were as follows: initial denaturation at 94 °C for 2 min (1 cycle); 98 °C for 10 s and 68 °C for 20 min (30 cycles); and 6 a final extension at 8 °C for 10 min (1 cycle). After the amplification reaction was completed, PCR products were purified using 1×Ampure PB beads (Pacific Biosciences), quantified using the Qubit dsDNA BR assay kit (Thermo Fisher Scientific), and quality-checked by agarose gel electrophoresis. Crucially, the amplification success rate of LR-PCR across all 200 samples was 100%.

Single-molecule real-time (SMRT) sequencing libraries were constructed as previously described with minor modifications [19]. Briefly, end-repair and adapter ligation were performed to add unique barcode adapters to each purified PCR product. Each 10 µL reaction mixture contains 4 µL PCR products, 5 µmol/L barcode adapter (Integrated DNA Technologies), 1×T4 DNA ligase buffer (Enzymatics), 1 mmol/L ATP (New England Biolabs), 200 µmol/L dNTP (New England Biolabs), 2.5 units T4 polynucleotide kinase (Enzymatics), 0.75 units T4 DNA polymerase (Enzymatics), and 180 units T4 DNA ligase (HC) (Enzymatics). The reaction mixture was incubated at 37 °C for 20 min, 25 °C for 15 min, and 65 °C for 10 min. Subsequently, 0.5 µL exonuclease III (NEB, Cat#M0206L) and 0.5 µL exonuclease VII (NEB, Cat#M0379L) were added to remove unligated adapters, and the reaction was continued at 37 °C for 1 h. Purify the DNA twice using 0.6 × Ampure PB magnetic beads (PacBio, Cat#100 265 900) according to the kit instructions. Finally, elute the DNA with 10 µL of Elution Buffer, which is the target PacBio sequencing library. DNA concentration was determined using the Qubit dsDNA HS assay kit (ThermoFisher, Cat# Q32851) on a Qubit 3 Fluorometer (ThermoFisher, Cat#Q33216). Each sample was quantified in triplicate, and the average concentration (≥ 3 ng/µL) was used for downstream sequencing. Libraries were bound to the DNA polymerase using the Sequel Binding Kit 2.0 and Internal Control Kit 1.0 (PacBio, Cat#101-842-900) according to SMRT Link software calculations. Sequencing primer (PacBio, Cat#100-970-100) was added to generate the final polymerase–primer–template complex, which was loaded onto a SMRT Cell and sequenced on the Sequel II platform (Pacific Biosciences).

### Data processing and quality control

Circular Consensus Sequencing (CCS) reads were generated from the raw subreads in BAM files output by the PacBio Sequel II sequencing platform using CCS software (Pacific Biosciences). The CCS reads were demultiplexed and trimmed using the lima software in the Pbbioconda software package (Pacific Biosciences). Filtered CCS reads were then aligned to the human reference genome hg38 using the pbm2 software to specifically obtain the target *PAH* gene fragment. Single-nucleotide variants (SNVs) and small insertions/deletions (indels) were called using FreeBayes 1.3.4. Variant pathogenicity was annotated following the guidelines of the American College of Medical Genetics and Genomics (ACMG), with reference to the ClinVar and Human Gene Mutation Database (HGMD) [20]. Haplotype analysis was performed using the pbampliconclustering software (Pacific Biosciences; https://github.com/PacificBiosciences/pbampliconclustering). Identified variants were visualized using the Integrative Genomics Viewer (IGV). For quality control, each *PAH* amplicon was required to achieve ≥ 30 CCS reads per sample to ensure sufficient coverage for variant analysis.

### Statistical analysis

All data were recorded and managed using Microsoft Excel. The traditional PKU screening workflow was compared with the optimized CAPAH-based process across three key indicators: missed detection rate (proportion of cases negative by traditional biochemical or genetic methods but positive by CAPAH), recall rate (proportion of newborns requiring recall for rescreening), and diagnostic turnaround time (interval from initial positive screening to definitive diagnosis). Associations among *PAH* variant types, variant spectrum, and genotype-phenotype correlations were also analyzed. Statistical significance was evaluated using paired t-tests or Wilcoxon signed-rank tests, with *p* < 0.05 considered statistically significant.

## Results

### Development and validation of CAPAH

To evaluate the accuracy of CAPAH in detecting *PAH* variants, we compared the results obtained from 20 DNA samples previously genotyped by NGS, MLPA, and Sanger sequencing. Among these samples, two harbored homozygous variants, one carried three heterozygous variants, one carried both a point variant and a large deletion, and the remaining 16 were compound heterozygotes for point variants (Table 1). These results showed that CAPAH achieved 100% concordance with conventional methods in all 20 cases. Notably, CAPAH enabled direct determination of the cis-trans relationships of multiple variants within the same amplified fragment (YZ02, YZ03) without requiring parental information (Fig. 1B and C), and precisely determined breakpoints of a large deletion (YZ06) (Fig. 1D).

**Table 1:** Validation of the CAPAH assay using samples with known *PAH* genotypes

Sample	Phenotype	NGS + MLPA + Sanger Sequencing	CAPAH	Concordant	Special Notes
YZ01	cPKU	c.728G > A, homo	c.728G > A, homo	Yes	-
YZ02	cPKU	c.977G > A/c.1199G > C	c.977G > A/c.1199G > C	Yes	Determine the *cis*/*trans* relationship of variants without pedigree analysis
YZ03	cPKU	c.722delG/c.907delT	c.722delG/c.907delT	Yes	Determine the *cis*/*trans* relationship of variants without pedigree analysis
YZ04	cPKU	c.331 C > T/c.728G > A	c.331 C > T/c.728G > A	Yes	-
YZ05	cPKU	c.194T > C/c.1238G > C	c.194T > C/c.1238G > C	Yes	-
YZ06	MHP	c.782G > A/Deletion	c.782G > A/c.-4163_-406del3758	Yes	Directly determine the breakpoints of large fragment deletions
YZ07	MHP	c.331 C > T/c.1174T > A	c.331 C > T/c.1174T > A	Yes	-
YZ08	MHP	c.688G > A, homo	c.688G > A, homo	Yes	-
YZ09	MHP	c.158G > A/c.442-1G > A	c.158G > A/c.442-1G > A	Yes	-
YZ10	MHP	c.158G > A/c.1197 A > T	c.158G > A/c.1197 A > T	Yes	-
YZ11	MHP	c.208_210del/c.355 C > T	c.208_210del/c.355 C > T	Yes	-
YZ12	MHP	c.466G > C/c.688G > A	c.466G > C/c.688G > A	Yes	-
YZ13	MHP	c.782G > A/c.929 C > G	c.782G > A/c.929 C > G	Yes	-
YZ14	MHP	c.158G > A, c.842 + 2T > A/c.1237 C > T	c.158G > A, c.842 + 2T > A/c.1237 C > T	Yes	-
YZ15	MHP	c.158G > A/c.611 A > G	c.158G > A/c.611 A > G	Yes	-
YZ16	MHP	c.158G > A/c.728G > A	c.158G > A/c.728G > A	Yes	-
YZ17	MHP	c.158G > A/c.1238G > C	c.158G > A/c.1238G > C	Yes	-
YZ18	MHP	c.158G > A/c.694 C > T	c.158G > A/c.694 C > T	Yes	-
YZ19	MHP	c.158G > A/c.782G > A	c.158G > A/c.782G > A	Yes	-
YZ20	MHP	c.158G > A/c.1199 + 1G > C	c.158G > A/c.1199 + 1G > C	Yes	-

cPKU, classic PKU; MHP, mild HPA

### Application of CAPAH in second-tier screening for PKU in newborns

A retrospective analysis was conducted on 200 newborn samples using CAPAH. These samples included 75 samples with borderline results in the initial biochemical screening (blood Phe concentrations: 1.6-2 mg/dL) and 125 samples with positive results (blood Phe concentrations > 2 mg/dL) (Table 2). CAPAH identified 97 carriers/negative individuals, thereby reducing unnecessary recalls by 48.5% (97/200). Among newborns who were negative upon rescreening (*n* = 75) and those who were rescreen-positive but later excluded (*n* = 56), CAPAH detected compound heterozygous variants in 10 and 30 newborns, respectively. This demonstrates that CAPAH can identify approximately 20% (40/200) of compound heterozygous cases that were overlooked by conventional screening, regardless of their ultimate clinical phenotype. CAPAH results were fully consistent with those from 59 patients confirmed by traditional sequencing (NGS + MLPA + Sanger sequencing). Importantly, among seven newborns previously classified as carriers by NGS, CAPAH revealed one (variant c.442-1G > A) to be homozygous, consistent with both Sanger sequencing (Fig. 1E) and the newborn’s clinical presentation (initial blood Phe: 9.25 mg/dL; rescreening: 26.19 mg/dL), leading to a diagnosis of classic PKU. Furthermore, in cases where a proband carried a de novo variant or when the variants carried by the proband are carried by both parents, conventional sequencing could not determine the phase of the variants. In contrast, CAPAH unambiguously demonstrated the trans configuration (Fig. 1F-H).

**Table 2:** Concordance between CAPAH and conventional PCR-based assays in identifying pathogenic *PAH* variants in newborn DBS

Initial screening of Phe (mg/dL)	No.	Second-tier screening of Phe (mg/dL)	No.	Proportion	Clinical diagnosis	No.	Proportion	Traditional sequencing^*^	No.	Proportion	CAPAH	No.	Proportion	Comments
Positive/ borderline (> 1.6)	200	Negative (0.37-2)	75	37.50%	ND	NA	NA	ND	NA	NA	compound heterozygous cases	10	13.33%	Traditional screening: missed detections.
											Carriers	25	33.33%	CAPAH applied to the second-tier screening: avoid recalls.
											Negative subjects	40	53.33%	
		Positive (> 2)	125	62.50%	No	56	44.80%	ND	NA	NA	compound heterozygous cases	30	53.57%	Traditional screening: missed detections.
											Carriers	16	28.57%	CAPAH applied to the second-tier screening: avoid recalls.
											Negative	10	17.86%	
					Yes	69	55.20%	Patients	59	85.51%	Patients (compound heterozygous cases)	59	85.51%	Traditional sequencing and CAPAH: 100% consistent.
								Patients/ Carriers	3	4.35%	Patients (compound heterozygous cases)	3	4.35%	Variants exist in both parents (*n* = 1) & de novo variants (*n* = 2) Traditional screening: cannot determine the cis-trans configuration. CAPAH: can prove the trans arrangement.
								Carriers	7	10.14%	Patients (compound heterozygous cases)	1	1.45%	CAPAH corrects the genotypes detected by NGS (heterozygous-homozygous).
											Carriers	6	8.70%	CAPAH applied to the second-tier screening: avoid recalls.

ND, not done; NA, not applicable

^*^Traditional sequencing: NGS for newborn, Sanger sequencing for pedigree analysis

### Spectrum of *PAH* variants detected by CAPAH

Among the 200 retrospectively analyzed newborns, CAPAH identified 103 newborns with biallelic variants, 47 carriers, and 50 negative individuals. In the 103 newborns with biallelic variants, a total of 64 *PAH* variants were detected, comprising 47 missense, eight splice-site, four nonsense, and five deletion variants (one large and four small fragment deletions). Variants were distributed across nearly all exons, except exons 9 and 13 (Fig. S2). The most frequently detected variants were c.158G > A (exon 2), c.728G > A (exon 7), and c.688G > A (exon 6), which were observed 51, 23, and 17 times, respectively (Table 3). Notably, 36 variants (56.25%) were detected only once, reflecting a high level of genetic heterogeneity in the cohort.

**Table 3:** Frequency and distribution of *PAH* variants among 103 newborns with biallelic variants

Changes in bases	Changes in amino acids	Variant sites	variant types	Allele numbers	Relative frequencies (%)	Phenotype	Simplified phenotype
c.158G > A	p.Arg53His	Exon 2	Missense	51	24.76%	MHP	MHP
c.728G > A	p.Arg243Gln	Exon 7	Missense	23	11.17%	cPKU	PKU
c.688G > A	p.Val230Ile	Exon 6	Missense	17	8.25%	MHP	MHP
c.442-1G > A	-	Intron 4	Splicing	8	3.88%	cPKU	PKU
c.1174T > A	p.Phe392Ile	Exon 11	Missense	7	3.40%	MHP	MHP
c.1238G > C	p.Arg413Pro	Exon 12	Missense	7	3.40%	cPKU	PKU
c.611 A > G	p.Tyr204Cys	Exon 6	Missense	6	2.91%	cPKU	PKU
c.739G > C	p.Gly247Arg	Exon 7	Missense	4	1.94%	cPKU	PKU
c.782G > A	p.Arg261Gln	Exon 7	Missense	4	1.94%	cPKU	PKU
c.842 + 2T > A	-	Intron 7	Splicing	4	1.94%	cPKU	PKU
c.898G > T	p.Ala300Ser	Exon 8	Missense	4	1.94%	MHP	MHP
c.1068 C > A	p.Tyr356*	Exon 11	Nonsense	4	1.94%	cPKU	PKU
c.320 A > G	p.His107Arg	Exon 3	Missense	3	1.46%	MHP	MHP
c.740G > T	p.Gly247Val	Exon 7	Missense	3	1.46%	cPKU	PKU
c.1123 C > G	p.Gln375Glu	Exon 11	Missense	3	1.46%	mPKU	PKU
c.1256 A > G	p.Gln419Arg	Exon 12	Missense	3	1.46%	mPKU	PKU
c.200 C > G	p.Ser67Cys	Exon 3	Missense	2	0.97%	not classified	not classified
c.311 C > A	p.Ala104Asp	Exon 3	Missense	2	0.97%	cPKU-mPKU	PKU
c.482T > C	p.Phe161Ser	Exon 5	Missense	2	0.97%	cPKU	PKU
c.532G > A	p.Glu178Lys	Exon 6	Missense	2	0.97%	not classified	not classified
c.722G > A	p.Arg241His	Exon 7	Missense	2	0.97%	mPKU	PKU
c.764T > C	p.Leu255Ser	Exon 7	Missense	2	0.97%	cPKU	PKU
c.977G > A	p.Trp326*	Exon 10	Nonsense	2	0.97%	cPKU	PKU
c.1162G > A	p.Val388Met	Exon 11	Missense	2	0.97%	cPKU	PKU
c.1222 C > T	p.Arg408Trp	Exon 12	Missense	2	0.97%	cPKU	PKU
c.1262T > C	p.Ile421Thr	Exon 12	Missense	2	0.97%	mPKU	PKU
c.1301 C > A	p.Ala434Asp	Exon 12	Missense	2	0.97%	cPKU	PKU
c.1316-1G > A	-	Intron 12	Splicing	2	0.97%	cPKU	PKU
c.-4163_-406del3758	deletion 3758 bp	Exon 1	Deletion	1	0.49%	not classified	not classified
c.47_48delCT	del 2 bp codon 15	Exon 1	Deletion	1	0.49%	cPKU	PKU
c.116T > C	p.Phe39Ser	Exon 2	Missense	1	0.49%	not classified	not classified
c.151G > C	p.Val51Leu	Exon 2	Missense	1	0.49%	not classified	not classified
c.158G > T	p.Arg53Leu	Exon 2	Missense	1	0.49%	not classified	not classified
c.208_210delTCT	del 3 bp codon 70	Exon 3	Deletion	1	0.49%	mPKU	PKU
c.212G > A	p.Arg71His	Exon 3	Missense	1	0.49%	MHP	MHP
c.242 C > A	p.Thr81Asn	Exon 3	Missense	1	0.49%	not classified	not classified
c.284_286delTCA	del 3 bp codon 94	Exon 3	Deletion	1	0.49%	cPKU	PKU
c.355 C > T	p.Pro119Ser	Exon 4	Missense	1	0.49%	mPKU	PKU
c.441 + 5G > T	-	Intron 4	Splicing	1	0.49%	cPKU	PKU
c.446T > C	p.Phe149Ser	Exon 5	Missense	1	0.49%	not classified	not classified
c.498 C > G	p.Tyr166*	Exon 5	Nonsense	1	0.49%	cPKU	PKU
c.505 C > A	p.Arg169Ser	Exon 5	Missense	1	0.49%	MHP	MHP
c.510T > A	p.His170Gln	Exon 6	Missense	1	0.49%	cPKU	PKU
c.516G > T	p.Gln172His	Exon 6	Missense	1	0.49%	not classified	not classified
c.574 A > G	p.Lys192Glu	Exon 6	Missense	1	0.49%	not classified	not classified
c.617 A > G	p.Tyr206Cys	Exon 6	Missense	1	0.49%	cPKU	PKU
c.689T > C	p.Val230Ala	Exon 6	Missense	1	0.49%	mPKU	PKU
c.707-1G > A	-	Intron 6	Splicing	1	0.49%	cPKU	PKU
c.721 C > T	p.Arg241Cys	Exon 7	Missense	1	0.49%	mPKU	PKU
c.722delG	del 1 bp codon 241	Exon 7	Deletion	1	0.49%	cPKU	PKU
c.755G > C	p.Arg252Pro	Exon 7	Missense	1	0.49%	cPKU	PKU
c.811 C > T	p.His271Tyr	Exon 7	Missense	1	0.49%	not classified	not classified
c.833 C > T	p.Thr278Ile	Exon 7	Missense	1	0.49%	cPKU	PKU
c.838G > A	p.Glu280Lys	Exon 7	Missense	1	0.49%	cPKU	PKU
c.975 C > G	p.Tyr325*	Exon 10	Nonsense	1	0.49%	cPKU	PKU
c.1139 C > T	p.Thr380Met	Exon 11	Missense	1	0.49%	MHP	MHP
c.1194 A > G	p.Lys398Lys	Exon 11	Splicing	1	0.49%	cPKU	PKU
c.1197 A > T	p.Val399Val	Exon 11	Splicing	1	0.49%	cPKU	PKU
c.1199G > A	p.Arg400Lys	Exon 11	Missense	1	0.49%	cPKU-mPKU	PKU
c.1199G > C	p.Arg400Thr	Exon 11	Missense	1	0.49%	cPKU	PKU
c.1200–2 A > C	-	Intron 11	Splicing	1	0.49%	cPKU	PKU
c.1208 C > T	p.Ala403Val	Exon 12	Missense	1	0.49%	MHP	MHP
c.1252 A > C	p.Thr418Pro	Exon 12	Missense	1	0.49%	mPKU	PKU
c.1303G > A	p.Asp435Asn	Exon 12	Missense	1	0.49%	not classified	not classified

cPKU, classic PKU; mPKU, mild PKU; MHP, mild HPA

### Genotype-phenotype distribution

**Fig. 2:**
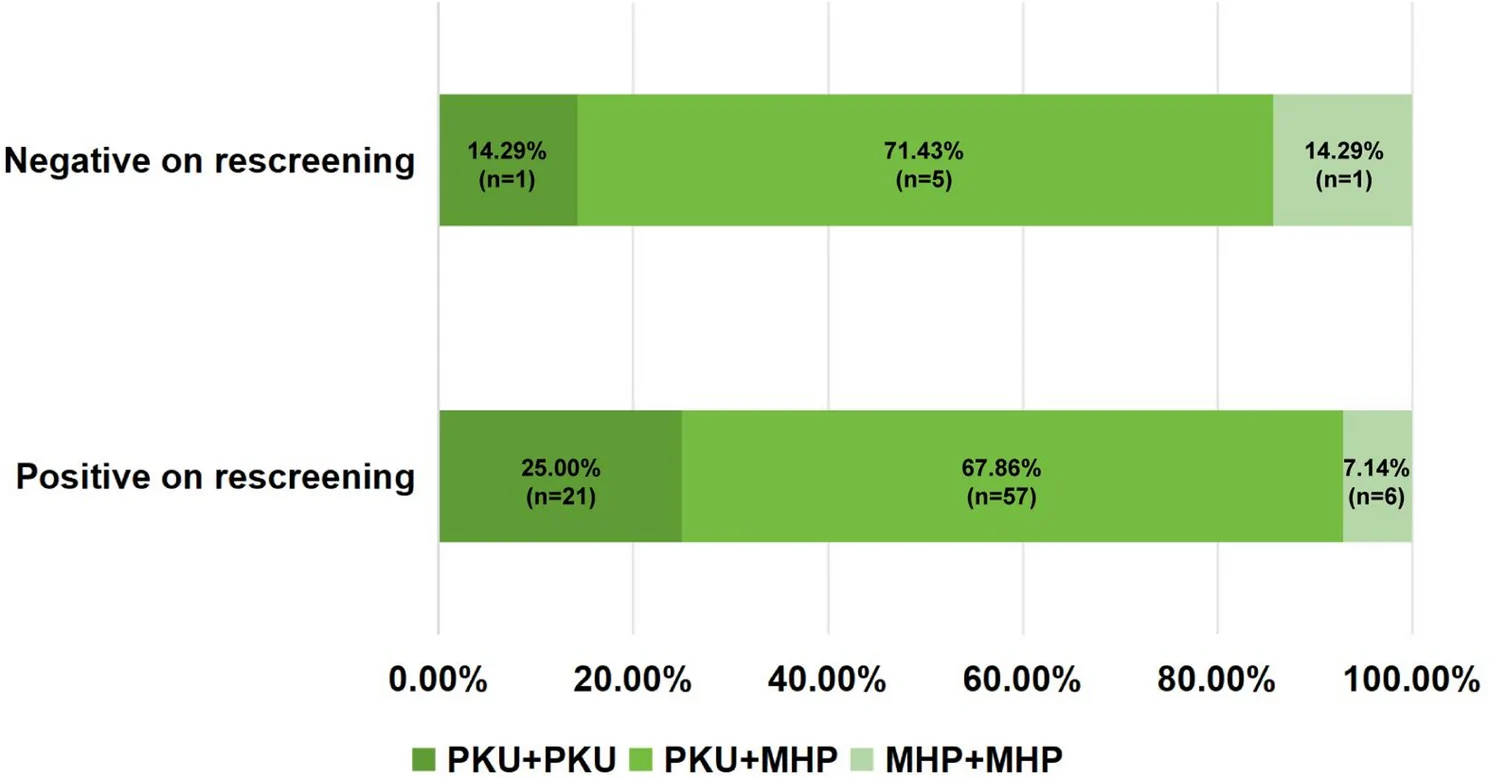
Distribution of neonatal *PAH* genotypes in the rescreening-negative group and the rescreening-positive group

Based on blood Phe concentrations, PKU can be clinically classified into three subtypes: classic PKU (cPKU, Phe: ≥ 20 mg/dL), mild PKU (mPKU, Phe: 6–20 mg/dL), and mild hyperphenylalaninemia (MHP, Phe: 2–6 mg/dL) [5, 21]. According to Garbade et al. (2019) and the *PAH*vdb database (https://www.biopku.org/home/pah.asp), each *PAH* variant was assigned an allelic phenotype value (APV) representing its predicted clinical severity [22]. Among the 103 newborns with biallelic variants identified by CAPAH, a total of 64 *PAH* variants were identified, including 32 cPKU-related, two cPKU/mPKU-overlapping, nine mPKU, nine MHP, and 12 unclassified variants (Table 3). After excluding cases with unclassified variants (*n* = 12), 91 genotype-positive newborns were included in the genotype-phenotype correlation analysis. For simplicity, cPKU and mPKU variants (both requiring clinical intervention) were categorized as PKU-severity variants. In the rescreen-negative group (*n* = 7), one patient carried two PKU-related variants, five carried one PKU- and one MHP-related variant, and one carried two MHP-related variants. In contrast, in the rescreen-positive group (*n* = 84), 21 genotype-positive newborns carried two PKU-related variants, 57 carried one PKU- and one MHP-related variant, and six carried two MHP-related variants. Compared with the rescreen-negative group, the rescreen-positive group exhibited a higher prevalence of biallelic PKU-related variants (25.00% vs. 14.29%), and a lower prevalence of dual MHP-related variants (7.14% vs. 14.29%) (Fig. 2), suggesting a strong correlation between variant severity and biochemical phenotype.

### Analysis of the variant of uncertain significance (VUS) c.158G > A

Among the 103 newborns with biallelic variants identified by CAPAH, 49 carried the VUS variant c.158G > A. After excluding cases with unclassified variants (*n* = 3) or those harboring additional compound heterozygous variants (*n* = 5), 41 newborns were included for further analysis. One newborns were homozygous for c.158G > A (Group 1), with the initial and rescreening blood Phe levels of 2.05 mg/dL and 2.07 mg/dL respectively. Thirty-eight newborns were compound heterozygous for c.158G > A and PKU-related pathogenic variants (Group 2), with mean initial and rescreening blood Phe levels of 2.11 ± 0.36 mg/dL and 2.40 ± 0.36 mg/dL, respectively. And the rescreening level significantly increased (*p* < 0.0001 by both paired t-test and Wilcoxon test). Two newborns were compound heterozygous for c.158G > A and MHP-related variants (Group 3), with mean initial and rescreening blood Phe levels of 2.11 ± 0.30 mg/dL and 2.25 ± 0.30 mg/dL, respectively (Table 4). Across all groups, Phe levels remained within the MHP range, though three newborns in Group 2 had rescreening Phe concentrations exceeding 3.0 mg/dL (3.05, 3.13 and 3.40 mg/dL). These findings suggest that c.158G > A may exert a mild functional impact on PAH activity, particularly when combined with pathogenic variants.

**Table 4:** Initial and second-tier screening results of Phe concentrations in newborns carrying the *PAH* c.158G > A variant

Group	Genotype	Number of cases	Proportion	Initial screening of Phe (mg/dL)	Second-tier screening of Phe (mg/dL)	*p* value (t test)	*p* value (Wilcoxon signed-rank test)
Group 1	c.158G > A, homozygous	1	2.44%	2.05	2.07	-	-
Group 2	c.158G > A + PKU, compound heterozygous	38	92.68%	2.11 ± 0.36 (1.64–3.29)	2.40 ± 0.36 (1.91–3.40)	1.88 × 10 − 5	2.87 × 10⁻⁵
Group 3	c.158G > A + MHP, compound heterozygous	2	4.88%	2.11 ± 0.30 (1.89–2.32)	2.25 ± 0.30 (2.04–2.06)	-	-
-	Total	41	100.00%	2.15 ± 0.39 (1.64–3.29)	2.37 ± 0.37 (1.46–3.40)	-	-

## Discussion

In this study, we developed and validated a full-length *PAH* gene sequencing method (CAPAH) based on the PacBio LRS platform and applied it, for the first time, in second-tier screening for PKU in newborns. CAPAH achieved 100% concordance with conventional sequencing methods in samples with known genotypes and demonstrated excellent variant detection and classification performance in real-world clinical specimens, thereby improving diagnostic accuracy and reducing unnecessary recalls.

First, CAPAH proved to be a highly reliable and accurate tool for comprehensive *PAH* genotyping. In 20 validation samples with known genotypes, CAPAH results were fully consistent with those obtained using traditional methods, including NGS, MLPA, and Sanger sequencing, confirming the reliability of the CAPAH test results. In complex situations involving multiple coexisting variants (in the same amplified fragment) and large deletions, CAPAH was able to determine the cis-trans relationship of variants and accurately define deletion breakpoints, capabilities that traditional methods typically lack. CAPAH can complete comprehensive testing for point variants, SNV/indels, and large deletions through a single PacBio platform, suggesting its unique value and clinical practicality in analyzing structurally complex regions and compound heterozygous variants.

Second, a retrospective analysis of 200 newborns with positive or borderline initial biochemical screening results further demonstrated the clinical utility of CAPAH. In conventional workflows, newborns with elevated Phe levels must undergo multiple steps, including recall and Phe concentration rescreening, biochemical tests, and genetic tests, before a definitive PKU diagnosis can be established. This process is often prolonged, in this study, the primary recall phase alone required 1 to 77 days (mean: 13 days; median: 10 days) and associated with high recall rates (Fig. S3, left). Subsequent confirmatory biochemical analyses typically took up to 10 days, while standard genetic testing required an additional 20 days, resulting in a lengthy overall diagnostic cycle with a historical median of 40 days. CAPAH-based second-tier screening reduced unnecessary recalls by approximately 48.5% (97/200) through direct genotyping, while avoiding about 20% of potential missed detections. By effectively bypassing most of the multi-step biochemical verification layers, CAPAH achieves an all-in-one molecular testing within approximately 14 days (Fig. S3, right), thereby substantially shortening the diagnostic cycle by a median of 26 days and alleviating parental anxiety and economic burdens. Moreover, the cost of CAPAH is estimated to be approximately $20 per sample. Under tranditional diagnostic strategies, the costs are approximately $25 for NGS [23], $24 for MLPA [24], $30 for Sanger sequencing [24]. Consequently, the cumulative expense for complex cases requiring multi-platform validation could escalate up to $79. Notably, newborns with borderline Phe concentrations (1.6-2.0 mg/dL) represent one of the most challenging groups in PKU screening. Their Phe levels often fall within a diagnostic gray zone, where conventional rescreening and biochemical assays frequently yield inconclusive results. CAPAH identified compound heterozygous *PAH* variants even in several rescreen-negative or clinically excluded cases, providing direct molecular evidence for newborns with ambiguous biochemical results. Thus, CAPAH not only reduces unnecessary recalls caused by false positives but also lowers the risk of missed detections among newborns with borderline values, addressing a major limitation of traditional biochemical screening.

Third, CAPAH provides new insights into genotype-phenotype correlations. The rescreening outcomes of newborns with positive or borderline initial screening were highly consistent with their genotypes. Biallelic PKU-related variants were significantly more frequent in the rescreen-positive group than in the rescreen-negative group, suggesting that persistently elevated Phe levels are more likely associated with a severe PKU genotype. In contrast, the majority of the rescreen-negative group carried PKU + MHP or double MHP-related variants. MHP-related variants often result in elevated residual enzyme activity, manifesting as mild or transient hyperphenylalaninemia, which may return to normal Phe levels on rescreening. These findings underscore that a single elevated Phe level on the initial screening test is insufficient to accurately distinguish PKU from MHP or may be a transient false positive. Routine rescreening remains essential for newborns with positive or borderline initial screening results. In cases with positive initial screening, integrating CAPAH can help achieve genotypic stratification of newborns at an early stage, assisting doctors in differentiating newborns requiring immediate intervention from those requiring dynamic observation, and promoting precise newborn screening follow-up and individualized management. Additionally, this study found that newborns compound heterozygous for c.158G > A and a PKU-related variant exhibited markedly elevated Phe levels during rescreening, suggesting that this genotype may have a higher metabolic risk and may present with more obvious phenotypic characteristics during rescreening. In contrast, newborns with homozygous c.158G > A or compound heterozygous with an MHP-related variant did not show significant differences during rescreening, suggesting that their metabolic status is relatively stable, possibly related to the relatively mild pathogenicity of the variant. Notably, the pathogenicity of the c.158G > A variant has not yet been fully elucidated. In vitro studies have shown that the c.158G > A variant can reduce PAH activity to approximately 79% of the wild-type levels [25, 26]. Wang et al. monitored two newborns carrying the compound heterozygous variants c.158G > A/c.611 A > G (cPKU) and c.158G > A/c.1238G > C (cPKU) for approximately one year (with initial Phe screening concentrations of 1.58 mg/dL and 2.04 mg/dL, respectively). They found that both newborns could tolerate a normal diet, and their blood Phe levels remained within normal ranges during follow-up. The mother of one of the newborns was homozygous for the c.158G > A variant and had not received a low-protein, low-Phe diet intervention for a long time. Her blood Phe concentration and Phe/tyrosine ratio remained within the normal range. Therefore, it is speculated that the reduction in enzyme activity caused by this variant is not sufficient to cause clinical symptoms of PKU, and is recommended to be classified as “likely benign” [27]. However, in our study, c.158G > A exhibited differential responses in different combinations: blood Phe levels were significantly elevated when combined with a PKU variant, whereas they remained within the MHP range when combined with an MHP variant or were homozygous. This suggests that c.158G > A may exert a relatively mild functional impact, although robust in vitro or in vivo functional evidence to confirm its pathogenicity has not yet been established. Consequently, based on ACMG guidelines, this variant should be classified as a VUS in clinical screening practices, and its correlation with the mild HPA phenotype should be cautiously interpreted during genetic counseling, particularly when present in a compound heterozygous state. In this study, all newborns carrying c.158G > A had blood Phe concentrations below the threshold of 6 mg/dL typically required for immediate and strict dietary therapy [28]. Therefore, for newborns carrying this variant, dynamic changes in blood Phe levels during rescreening should be monitored to avoid underestimating risk due to initial screening results nearing the threshold. The focus of clinical management is long-term monitoring rather than emergency intervention. A standardized follow-up plan should be established to regularly monitor blood Phe concentrations and neuropsychological development. Awareness should be maintained of the potential for adverse outcomes in the minority of individuals with critically high Phe levels (e.g., persistently > 4 mg/dL), provide individualized nutritional guidance in a timely manner.

A total of 64 *PAH* variants were identified in this study. Among them, missense variants accounted for the majority (47/64, 73.4%), while splice-site, nonsense, and deletions were relatively rare. This distribution aligns with international databases such as ClinVar and HGMD, supporting the accuracy of CAPAH results. Variants were detected in all exons except exons 9 and 13, highlighting the widespread distribution of *PAH* variants and underscoring the necessity of a comprehensive full-length sequencing strategy. In terms of variant frequency, c.158G > A, c.728G > A, and c.688G > A were the most frequently detected variants in this cohort, suggesting that hotspot variants are specific to different regions or populations (this study was conducted on positive/borderline initial screening enrichment samples, not the general population or PKU patients) [29–31]. The identification of these variants not only helps to optimize regional rapid testing panels but also provides a basis for further investigation of genotype-phenotype relationships. Notably, more than half of the variants (36/64, 56.25%) occurred only in one newborn, suggesting a high degree of genetic heterogeneity in the PKU pathogenic spectrum. Different from the hotspot variants, these rare variants often lack sufficient clinical and functional evidence, and their pathogenicity must be classified based on the ACMG guidelines and subsequent functional studies, family verification, etc. ^20^. This also emphasizes the important value of CAPAH in unbiased detection of rare variants, avoiding the risk of missed detection when relying on limited panels or databases.

The clinical significance of this study lies in the fact that CAPAH, as a novel second-tier screening tool for PKU in newborns, can establish a direct molecular link between traditional screening and final diagnosis, shortening diagnostic time and improving the screening accuracy. In the current screening process, recall and confirmation stages often take weeks or even longer. The application of CAPAH has the potential to complete molecular diagnosis within two weeks, enabling some newborns to receive dietary intervention as soon as possible during the critical early window, thereby improving long-term outcomes. Nevertheless, this study still has certain limitations. First, the study was limited by its retrospective design, single-center nature, and potential selection bias. The 200 samples were selected from 2019 to 2024 based on initial Phe > 2 mg/dL (*n* = 125) or borderline 1.6-2 mg/dL (*n* = 75). Consequently, this represents an enriched cohort with a high pretest probability of carrying PAH variants, and the sample size for independent validation was modest (*n* = 20). Therefore, Therefore, the reported 48.5% reduction in recalls applies specifically to this enriched second-tier screening context and cannot be directly generalized to unselected primary screening newborn populations. Future large-scale, multi-center, and prospective studies are required to validate the effectiveness of CAPAH in real-world screening scenarios. Second, although CAPAH performs well in terms of detection rate and accuracy, its higher cost and operational complexity compared to NGS may limit widespread implementation, requiring further cost-effectiveness optimization. Third, while CAPAH effectively identifies newborns with molecularly confirmed bi-allelic variants, definitive clinical diagnosis strictly necessitates biochemical and phenotypic correlation. This study lacks comprehensive long-term follow-up data; hence, the current follow-up period was insufficient to evaluate long-term neurodevelopmental outcomes or fully characterize the clinical trajectories of asymptomatic individuals. Moreover, the functional significance of c.158G > A and several unclassified variants remains to be elucidated, and the current follow-up period was insufficient to evaluate long-term neurodevelopmental outcomes. Future work should include in vitro functional analyses and prospective, large-scale follow-up studies to further clarify variant pathogenicity and validate the clinical value of CAPAH in newborn PKU screening.

## Conclusion

In summary, this study demonstrated that CAPAH, a method developed based on the PacBio LRS platform, enables comprehensive and accurate detection of *PAH* variants. It not only efficiently identifies complex variants such as point variants, large deletions, and compound heterozygosities, but also directly determines cis-trans relationships. When applied to the second-tier screening for PKU in newborns, CAPAH markedly enhances diagnostic accuracy, shortens turnaround time, and reduces unnecessary recalls. In particular, for newborns with borderline Phe levels in the initial screening, CAPAH provides definitive molecular evidence, minimizing missed detections and repeated recalls. With further cost reductions and technological optimization, CAPAH is expected to become an important tool for precise screening and early diagnosis of PKU in newborns, providing crucial technical support for improving screening precision, establishing genotype–phenotype correlations, and facilitating accurate variant interpretation.

## Supplementary Information

Below is the link to the electronic supplementary material.

Supplementary Material 1

## Data Availability

The datasets used and/or analyzed during the current study are included within the article and its additional files.

## Abbreviations

PKU: Phenylketonuria
PAH: Phenylalanine hydroxylase
Phe: Phenylalanine
HPA: Hyperphenylalaninemia
DBS: Dried blood spots
BH4: Tetrahydrobiopterin
MLPA: Multiplex ligation-dependent probe amplification
NGS: Next-generation sequencing
qRT-PCR: quantitative real-time PCR
LRS: Long-read sequencing
LR-PCR: Long-fragment PCR
SMRT: Single-molecule real-time
CCS: Circular consensus sequencing
SNVs: Single nucleotide variations
indels: insertions and deletions
ACMG: American College of Medical Genetics and Genomics guidelines
HGMD: Human Gene Mutation Database
IGV: Integrative Genomics Viewer
cPKU: classic PKU
mPKU: mild PKU
MHP: Mild hyperphenylalaninemia
APV: Allelic phenotype value
VUS: Variants of uncertain significance
