# Supplementary material for: Development and clinical application of CAPAH: a long-read sequencing approach for accurate second-tier screening of phenylketonuria in newborns
Source: Orphanet J Rare Dis. 2026 Aug 12;21:407. doi: 10.1186/s13023-026-04537-6 (PMC13753362; doi:10.1186/s13023-026-04537-6)
Supplement: Supplementary file 1 [file 13023_2026_4537_MOESM1_ESM.docx]

**Supplementary Figures**

**Fig. S1. Agarose gel electrophoresis of genomic DNA and amplicons generated by long-range PCR via CAPKU from Cases 1-6.**

**Fig. S2.** **Scheme of detected *PAH* variants in this study.** Variants detected more than 10 times are indicated in blue.

**Fig. S3.** **Newborn PKU screening and diagnosis process.** Shown are the traditional process (left) and the process for applying CAPAH to second-tier screening for PKU in newborns (right).
